# Recent Progress on Piezoelectric and Triboelectric Energy Harvesters in Biomedical Systems

**DOI:** 10.1002/advs.201700029

**Published:** 2017-03-27

**Authors:** Qiang Zheng, Bojing Shi, Zhou Li, Zhong Lin Wang

**Affiliations:** ^1^ Beijing Institute of Nanoenergy and Nanosystems Chinese Academy of Sciences National Center for Nanoscience and Technology (NCNST) Beijing 100083 P. R. China; ^2^ School of Materials Science and Engineering Georgia Institute of Technology Atlanta GA 30332

**Keywords:** biomedicine, piezoelectric nanogenerators, self‐powered systems, triboelectric nanogenerators

## Abstract

Implantable medical devices (IMDs) have become indispensable medical tools for improving the quality of life and prolonging the patient's lifespan. The minimization and extension of lifetime are main challenges for the development of IMDs. Current innovative research on this topic is focused on internal charging using the energy generated by the physiological environment or natural body activity. To harvest biomechanical energy efficiently, piezoelectric and triboelectric energy harvesters with sophisticated structural and material design have been developed. Energy from body movement, muscle contraction/relaxation, cardiac/lung motions, and blood circulation is captured and used for powering medical devices. Other recent progress in this field includes using PENGs and TENGs for our cognition of the biological processes by biological pressure/strain sensing, or direct intervention of them for some special self‐powered treatments. Future opportunities lie in the fabrication of intelligent, flexible, stretchable, and/or fully biodegradable self‐powered medical systems for monitoring biological signals and treatment of various diseases in vitro and in vivo.

## 1 Introduction

Over last decades, implantable medical devices (IMDs) have experienced tremendous growth, becoming indispensable medical tools for improving the quality of life and prolonging the patient's lifespan. Currently, the IMDs have been implanted in various parts of human body as artificial treatments and diagnostic tools, including sensors, pacemakers, implantable cardioverter defibrillators, cochlear implant and stimulators for deep brain, nerve and bone.^1^ These implantable electronic devices can provide diagnosis (e.g. heart rate, blood pressure and temperature monitoring) for a number of diseases related to the heart, brain and some other important organs and support real‐time treatment (e.g. stimulation of muscle and nerve system). For instance, a cardiac pacemaker can help to correct abnormal heart rhythms by using electrical stimulation to contract the patients' cardiac muscle to relieve heart blockage or sick sinus syndrome.^2^ Additionally, to achieve better quality of life and enhance the survival rateof patients worldwide, IMDs have also contributed significantly to our cognition of the biological processes existing in the human body, including the complicated mechanisms of neural communication, memory and control, which significantly deepen our understanding of how these processes are affected by differernt diseases and treatments.^3^

In spite of substantial advancement in the manufacture and application of IMDs since the first in vivo cardiac pacemaker borned in 1958, the present IMDs are still faced with numerous challenges.^4^ There is a strong demand to design IMDs with diminishing size and weight for the sake of minimize their impact on daily human activities and increase comfort for the users. The batteries usually occupy the weight and size of the IMDs. However, they are limited by current technology and difficult to achieve miniaturization and weight reduction. The lifetime of battery is another challenge need to be overcome. When used to generate electrical pulse in deep brain stimulators (DBS) and cardiac pacemakers, the lifetime of those batteries is predetermined (e.g. 3 to 5 years for DBS), at the end of which these IMDs have to be replaced by surgery, leading to high cost of money to the patients and the social healthcare system.^5^

In vivo energy generation and internal charging by the natural body activity and physiological environment has been reported recently. Further miniaturization can be realized by means of self‐powered implantable devices that harvesting energy from natural sources or artificial power around the patient for sustaining the device directly. Variouse methods to reclaim energy from electrical, thermal, chemical and mechanical processes in vivo has been demonstrated, for example electric potentials from inner ear, glucose oxidation, vibration of organs and muscle contraction.^6^

In all these biological power sources, mechanical energy was considered as one of the most popular and sufficient power in a living creature. By harvesting in vitro and in vivo biomechanical energies, self‐powered medical electronics for nearly lifetime can be attained. Attractive approaches of self‐powered biomedical systems have been recently investigated by integration of energy harvesting devices, which can convert biomechanical energy from movements of human body (including body motion, blood circulation and the contraction/relaxation of cardiac, lung and muscle) into electricity.^7^ In this mechanical energy conversion process, many techniques such as piezoelectric effect, triboelectric effect, magnetostrictive effect and electromagnetic induction, can be used.^8^ However, another type of bulky energy‐harvesters utilized for implantable energy sources were limited, due to discrepant contact with the curved, corrugated and irregularly shaped surfaces of organs such as the lung, brain, eye and heart. Moreover, energy harvesters on rigid and thick substrates are unsuitable for converting the subtle movements of internal musclesand organs to generate electric power.

Extremely flexible and lightweight energy harvesters are settled conformally on muscle and organ surfaces. These devices are fabricated on plastic thin films such as polyethylene terephthalate (PET), polyimide (PI), and polydimethylsiloxane (PDMS) widely used in flexible electronics because of their appropriate flexibility and strength.

Recent development in biomechanical energy harvesters based on piezoelectric effect is an significant progress for solving the aforementioned issues. Several researchers have reported thier pliable piezoelectric energy harvesters with high performance called piezoelectric nanogenerators (PENGs),^9^ which use organic or inorganic materials such as polyvinylidene fluoride (PVDF),^10^ poly(vinylidenefluorideco‐trifluoroethylene) (P(VDF‐TrFE)),^11^ ZnO,^12^ BaTiO_3_(BTO),^13^ Pb(Zr_x_Ti_1–x_)O_3_(PZT),^14^ and (1–x) Pb(Mg_1/3_Nb_2/3_)O_3_‐xPbTiO_3_ (PMN‐PT).^15^ These functional devices can generate electric power under tiny irregular deformation and mechanical vibration revealing a tremendous potential to be applied in differernt medical devices in vivo. The fast growing triboelectric energy conversion devices, defined as triboelectric nanogenerator (TENG), have also shown many advantages, such as high efficiency, light‐weight, low costand easy fabrication, and provided new options for biomechanical energy harvesting.^16^ The mechanism of the TENG relies on a conjunction of triboelectrification and electrostatic induction between two contacted materials. Based on the triboelectric series,^17^ any two different materials with disparate tendency to lose or gain electrons in a frictional contact process have the potential to be used for TENG. Since there is a broad range of materials selections, we can take into account the output ability, flexibility, biocompatibility and cost of the device, which make TENG an ideal candidate for biomedical applications. Several research groups have employed high‐performance flexible and/or implantable TENGs for harvesting mechanical energy derived from the human motions or in vivo physiological movements. Together with the PENGs, TENGs are becoming most promising biomechanical energy harvester and providing great chance for building self‐powered biomedical systems.

**Figure 1:**
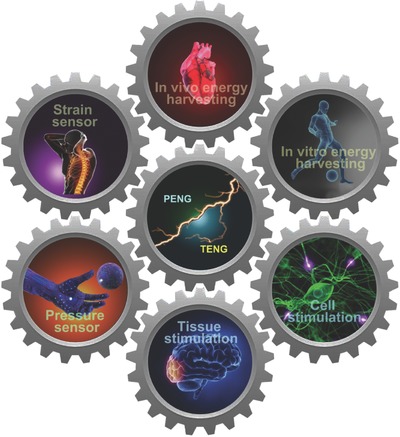
Recent applications of PENGs and TENGs in biomedical field.

Besides energy harvesters, PENG and TENG can be as mechanical nanosensors in biomedical field. By specific structural design, these devices can be very sensitive to detect small scale mechanical motions (e.g. the vibration of carotid artery caused by phonation and bloodstream). Recently, some studies reported the delicate biosensing applications of PENG and TENG. For example, piezoelectric device was reported as a electromechanical biosenser to monitor the volume change of PC12 cells and as bio‐inspired artificial hair cells to detect displacement of vibrational in 15 nm range.^18^ In addition, a triboelectric based bionic membrane sensor (BMS) can monitor the throat sound in high‐frequency and continuously detect the human arterial pulse wave in low‐frequency by one single device, exhibiting great potential in wearable medical/health monitoring.^19^ These progresses could be developed to real‐time sensing of cardiac function or blood pressure and in vivo monitoring the recovery of damaged sensorium in future. This review introduces a overview of recent progress of biomechanical energy harvesters, nanosensors and stimulator. Particularly, this manuscript presents applications of piezoelectric and triboelectric devices as energy harvester and self‐powered sensers according to their remarkable flexibility, biocompatibility and cost‐effective compared to other types of mechanical energy conversion means. Moreover, the applications of piezoelectric and triboelectric based devices in self‐powered cardiac pacemaker, nerves/muscles stimulator, and nanoscale sensor for monitoring biomedical pressure/strain changes, are discussed (**Figure** **1**).

## 2 Energy Conversion Mechanism

### 2.1 Piezoelectricity

Appling PENGs to harvest mechanical energy has received a great amount of attention due to its diversity of sophisticated design for direct conversion of mechanical energy into electric power for variouse integrated applications.

**Figure 2:**
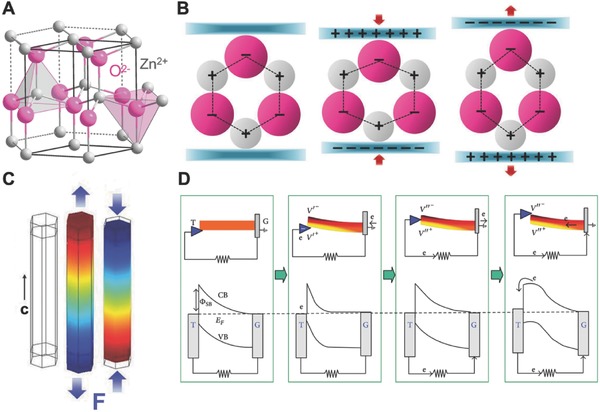
Mechanism of piezoelectricity. A) Atomic model of the wurtzite‐structured ZnO. B) Different piezopotential in tension and compression modes of the PENG. C) Numerical calculation of the piezoelectric potential distribution in a ZnO nanowire under axial strain. Reproduced with permission.[[qv: 20a]] Copyright 2009, AIP Publishing LLC. D) Band diagram for the charge outputting and flowing processes in the PENG. Reproduced with permission.[[qv: 20b]]

The nature of the piezoelectric effect is closely related to the generation of electric dipole moments in solids. The dipole moments may either be induced by ions on crystal lattice sites with asymmetric charge surroundings (as in ZnO, BaTiO_3_ and PZT) or may be carried by molecular groups directly (as in cane sugar). Take the ZnO crystal of wurtzite‐structured as example, the tetrahedrally coordinated Zn^2+^ and O^2−^ are accumulated layer‐by‐layer along the c‐axis (**Figure** **2**A). At its original state, the charge center of the anions and cations coincide with each another. Once applying an external force, the structure is deformed (stretching or compressing). Therefore, the negative and positive charge centers are separated and form an electric dipole leading to a piezoelectric potential (Figure 2B). If an external load is connected to the deformed material, the free electrons are driven to partially screen the piezoelectric potential and flow through the external circuit to realize a new equilibrium state.^20^ Therefore, a current pulse flowing through the external circuit is continuously generated when the piezoelectric potential is altered sequentially by applying a dynamic external force (Figure 2C,D).^21^ This primary mechanism of piezoelectric potential generation applies to various piezoelectric materials. PENGs mainly consist of piezoelectric materials and flexible substrates. Two core factors for developing PENG are material choosing and device structural design.

### 2.2 Triboelectricity & Electrostatic Induction

**Figure 3:**
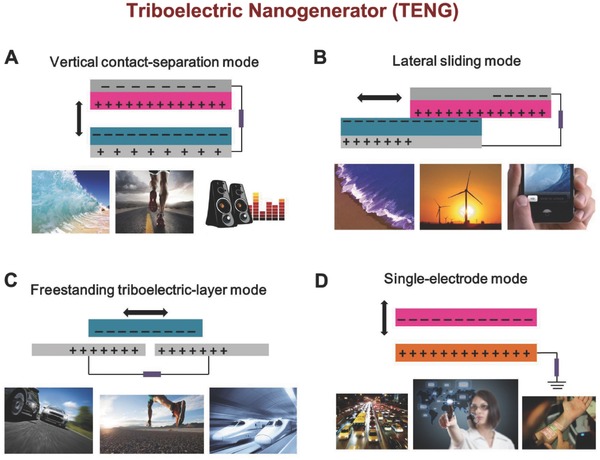
The four fundamental modes of TENGs: A) vertical contact‐separation mode; B) Lateral‐sliding mode; C) freestanding triboelectric‐layer mode, and D) single‐electrode mode.

The triboelectric effect which has been known for thousands of years, is the electrically charging process when two different materials contact each other through friction. Despite this is one of the most common phenomena that seen every day, the mechanism of triboelectrication is still not very clear. The process of chemical bonds formed between some parts of the surface in two different contacted materials and charges movinge from one material to another due to different stability to gain electron is generally believed. The charges transferred between two materials can be molecules, ions and electrons. When two materials are separated, some of the bonded atoms tend to maintain the additional transferred electrons, and some tend to give them away, which possibly give rise to the opposite charges on differernt friction materials. The opposite charges on both friction surfaces can generate a triboelectric potential, which can drive electrons in the back electrode to flow in order to balance the created electric potential drop. On the basis of this principle, four kinds of TENGs with different modes have been invented (**Figure** **3**), as elaborated in the following:

### 2.2.1 Vertical Contact‐Separation Mode

The first design of TENG was shown in Figure 3A as an example. Two differernt dielectric films place as top and bottom friction layer, and electrodes are deposited on outer surfaces of the friction film. The opposite charge is created between the surfaces of two contacted dielectric films. When the external force is released, the two dielectric films are separated with each other, accompanying with the electric potential drop between the friction surfaces. After connecting the two electrods with a load circuit, free electrons would flow from an electrode to another, driven by the electrostatic field. Once the gap becomes closed, the electric potential created by triboelectric effect disappears and the electrons flow backward.^22^

### 2.2.2 Lateral Sliding Mode

The original structure of lateral sliding mode and vertical contact‐separation mode have the same starting position. A relative parallel sliding creates triboelectric charges on the both surfaces, when two friction surfaces contacted (Figure 3B).^23^ Therefore the electrons on two electrodes are driven to flow by the triboelectric charges, which is generated by a lateral polarization introduced along the sliding direction. With the periodic process of sliding apart and closing, this lateral sliding mode TENG generates an AC output. The sliding can be a planar motion, a disc rotation^24^ or a cylindrical rotation. Related studies of mechanism have been used to improve the basic mode and grating structured TENG.

### 2.2.3 Single‐Electrode Mode

These two kinds of modes mentioned above contain two friction layers and two electrodes interconnected by an external circuit. Such TENGs can work independently and be freely moved. While in some other cases, a mobile object can be one electrod of the TENG, which do not need to be electrically connected to the load, such as a human jogging on the ground. For scavenging mechanical energy in these cases, single electrode TENG is created.The bottom electrode is grounded in single‐elecrtod mode TENG (Figure 3D). The local electrical field distribution will be changed with the top object approaching or departing from the bottom film, resulting in electron flow between the ground and the bottom electrode to match the potential change. In both contact‐sliding mode and contact‐separation mode, this energy harvesting strategy has been applied.^25^

### 2.2.4 Freestanding Triboelectric‐Layer Mode

In natural environment, a moving object, such as our cloths, gloves and shoes, is usually charged by contacting with air or ground. The electric charges can maintain on the surface for hours. Due to the density of electric charges achieves a maximum, there is no necessary to do friction or contact during this time. A pair of symmetric electrodes is designed under a dielectric film and the width of the electrodes and the gaps are of the same order with the moving object. With the object approaching to and departure of the electrodes, an asymmetric electric charge distribution on the surface of materials can be createed, which causes the electrons flowing from one electrodes to another to screen the local potential distribution (Figure 3C). Between the paired electrodes, an AC output is generated by the oscillation of the electrons. Because there is no direct friction or touching between the moving object and the top dielectric layer of the electrodes, free rotation is possible without direct mechanical contact, and abrasion of the dielectric layer can be completely reduced. This approach will grealy enhance the durability and prolong the service life of TENGs in rotation mode. Harvesting energy from human walking, automobile and other moving objects is feasible and convenient.^26^

## 3 Materials and Device Design

### 3.1 Nanowire Devices

The name of piezoelectric nanogenerators (PENGs) was defined by Wang's group in 2006 when they found piezoelectricity in ZnO nanowire.^9^ After that, numerous researches have been implemented about the working mechanism, structural design and modeling, and output optimization of the PENGs. Until recently, various kinds of flexible PENGs have been developed, which could be used for harvesting varies mechanical energies from either the environment or human bodies. The output electrical energy has been increased from several millivolts to several hundred volts, which is enough for driving a small devices with low power consumption, such as liquid crystal display (LCD), light‐emission diode (LED), and some wireless signal transmitting devices. In this section, the author briefly reviewed the working mechanism, modeling/simulations, and the experimental progress of piezoelectric nanogenerators according to the structure of the nanogenerators including the vertical aligned nanowire arrays, the lateral‐aligned nanowire networks, and some other similar structures, such as nanobelts and nanoribbons.

#### 3.1.1 Lateral Nanowires

**Figure 4:**
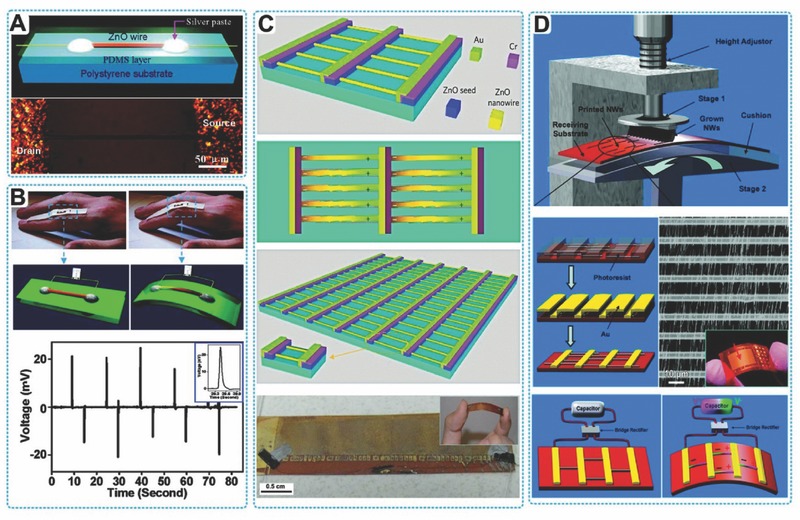
PENGs based on lateral ZnO nanowires. A,B) single nanowire based PENG fixed on flexible substrate. Reproduced with permission.^27^ Copyright 2009, Nature Publishing Group and American Chemical Society. C) Structure and optical images of a flexible lateral‐nanowire‐array integrated PENG. Reproduced with permission.^28^ Copyright 2010, Nature Publishing Group. D) Fabrication process of a flexible PENG based upon a lateral ZnO NW array. Reproduced with permission.^12^ Copyright 2010, American Chemical Society.

PENGs built with single ZnO nanowire or lateral nanowires arrays have been well established, providing various delicate nanoscale devices for multiple applications (**Figure** **4**). In a typical case, both ends of a single ZnO nanowire was fixed by silver paste as electrodes. The ZnO nanowire and two electrodes were packaged on a flexible substrate laterally. A uniaxial tensile strain was induced by the bending of the substrate and cause a piezoelectric potential along the ZnO nanowire, which drove the electrons to flow between the external circuit. A pulse current signal was generated by ZnO nanowire‐based PENG in a repeated bending‐releasing process (Figure 4A). However, the output performance of PENG based on single ZnO nanowire was relative low. The open‐circuit voltage (*V*_oc_) was about 20–50 mV and short‐circuit current (*I*_sc_) was about 400–750 pA, which limited their applications of energy harvesting (Figure 4B).^27^ For enhancing the output of ZnO based PENG, a direct approach was to integrate numerous lateral ZnO nanowires in a single device. As shown in Figure 4C, nearly 700 rows of ZnO nanowires were integrated on a single flexible substrate in a parallel configuration.^28^ Each row in this configuration contained about 20,000 ZnO nanowires. The whole device exhibited great flexibility. When this device was deformed by a linear motor at a straining rate of 2.13%, it reached about 1.2 V *V*_oc_ and 26 nA *I*_sc_ as an average, respectively. The slightly different of the magnitudes of the PENG output peaks is noted as the different straining rates in the releasing and stretching processes.

While for powering electrical devices, the mentioned output is still need to be improved and PENG with higher output is desired, which means the total number as well as the density of integrated ZnO nanowires need to be further increased. The lateral ZnO nanowire array was utilized in a sweeping‐printing method to fabricate high‐output PENG by Zhu et al. (Figure 4D).^12^ In a standard process, uniform ZnO nanowires were synthesized firstly on silicon substrates in physical vapor deposition (PVD) method. By sweeping on a flexible Kapton film, the vertically aligned nanowires were transferred to substrate surface by shear force. The avergae density of as‐transferred nanowires was 1.1 × 10^6^ cm^–2^. Next, 600 rows of Au electrodes in stripe‐shaped with space of 10 µm in width were deposited on top of the lateral nanowires arrays by conventional photolithographic procedures. At last, PDMS was used to package the entire device, which could further protect the device from invasive environment of humidity and corrosive chemicals, and enhance the mechanical robustness. The identical growth and alignment of nanowires insureed the direction of the piezoelectric potentials and a successful superposition effect of the electric energy generation of all integrated nanowires. It can reach up to 2.03 V of *V*_oc_ and an 11 mW cm^−3^ of output power density. Furthermore, the commercial LEDs were powered by the electrical energy generated by the PENG, demonstrating the great potential of PENG as a power source of small electronic devices. However, the photolithographic fabrication method limited the scale‐up and industrial production of this kind of PENG, for the process was technical, complex and expensive. To address this problem, a facile fabricating process of PENG was developed by coating conical ZnO nanowires into a flat and stretchable polymer film. This device generated a macroscopic electric potential by the superposition of piezoelectric potential in each conical nanowire between top and bottom electrodes. The generated energy was sufficient to drive a LCD and was reported as 2 V in voltage and 50 nA in current.^29^

Besides ZnO, many other nanowires made up of different materials also have shown great performance when fabricated into PENGs. Wu et al. described a series of work about robust and low cost PENG made by ZnSnO_3_ nano‐belts.^30^ One of the PENG was fabricated by the integration of thousands of randomly distributed lateral ZnSnO_3_ nanowires. Due to the spontaneous polarization of ZnSnO_3_ belts along the z‐axis [001], a substantial piezoelectric potential across the PENG's thickness was generated under a compressive strain of about 0.1% in the unipolar assembly of the triangular‐beltsdue to. A maximum output voltage and current reacheed up to 5.3 V and 0.13 µA, respectively.^31^ Dagdeviren and her co‐workers fabricated a conformal PENG based on lateral integrated PZT nano‐ribbons for harvesting mechanical energy from motions of the organs, such as diagrams, heartand lung. An output voltage of 4 V was achieved in this report, providing evidence that PENG can significantly yield electric energy from in vivo organs. Moreover, BaTiO_3_ nanowires synthesized via a simple and low temperature hydrothermal method were used to develop a lead‐free, flexible PENG by Park et al. During regular and periodical bending and releasing, the BaTiO_3_ nanowires–PDMS composite device successfully generated about 7.0 V of output voltageand 0.36 µA of output current.^32^

#### 3.1.2 Vertical Nanowires

**Figure 5:**
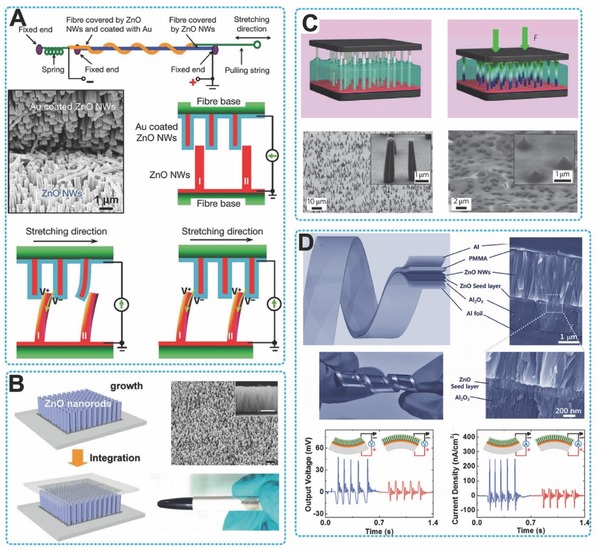
PENGs based on lateral ZnO nanowires. A) Experimental setup, internal structure, and mechanism of PENG fabricated by ZnO NWs vertically grown radially around textile fibers. Reproduced with permission.^36^ Copyright 2008, Nature Publishing Group. B) PENG prepared by growing ZnO NW arrays on graphene substrate. Reproduced with permission.^34^ C) PENG based on vertical ZnO nanowire arrays that grown by low‐temperature hydrothermal decomposition, which covered in PMMA by spin coating. Reproduced with permission.^28^ Copyright 2010, Nature Publishing Group. D) Super‐flexible ZnO based PENG with Al foil as both the substrate and the electrode. Reproduced with permission.^35^

Similar to the above mentioned lateral nanowires devices, the vertically grown ZnO nanowire arrays that have consistent polar directions can also be used to convert mechanical energy into electric power (**Figure** **5**). One of the most acceptable methods to fabricated vertical ZnO nanowires is solution‐based wet chemistry method (Figure 5C). The nanowire arrays can be synthesised at about 80 °C on various substrates. The uniformity and periodicity of these nanowires can be well controlled by pre‐sputtering a layer of seeds to the substrate.^33^ This technique provides a low cost, precticaland large scale approach to fabricate PENGs. In this context, a large number of flexible PENGs were developed, benefiting the design of self‐powered portable electronics. A fully flexible PENG was fabricated by Choi et al. based on vertically aligned ZnO nanowires in 2010.^34^ The working principle of this kind of PENGs was just similar with that of the former mentioned PENGs based on the lateral ZnO nanowires. The piezoelectric potential generated electron flow, which was controled by Schottky barrier between the interface of metal electrode and ZnO nanowires (Figure 5B). However, when built into a flexible device, the electrodes in this kind of energy harvester may appear some problems of mechanical durability, which make against the lifetime and stability of the device. Therefore, in the manuscript, they provided a potential solution by using single walled carbon nanotube (CNT) networksas conductive electrodes. Due to its improved contact between the nanowires and electrodes, the output of this PENG was significantly enhanced, and meanwhile its durability and stability was exceptional good compared with former devices. The same group further developed a flexible and transparent PENG consisting vertically grown ZnO nanowires using the conventional solution growth method and 2D graphene electrode. The 2D graphene electrode has exhibited extraordinary electrical and mechanical properties in comparison with other electrode materials, which made it an attractive candidate in the application of flexible electronics. Due to its outstanding properties, the as fabricated graphene‐based PENG showed a stable and reliable output current density of 2 µA cm^–2^. When the device was bended or rolled for several times, the output was without any fluctuations. The mechanical strength as well as its improved output performance provided this device a great potential for reliable biomedical applications. In 2011, Hu and her co‐workers reported a series work to demonstrate a new PENG fabricated with vertical ZnO nanowires arrays. An insulating layer of a thin layer of poly(methyl methacrylate) (PMMA) coated on top of the ZnO nanowires arrays was the most striking advance. The metal electrode was deposited on the PMMA layer. By coupling of the piezoelectric effect and the electrostatic induction, electron flows went back‐and‐forth in the external load induced by an iterative mechanical deformation applied on the device. The output voltage and the corresponding current were up to 20 V and 8 µA, respectively. However, this high output PENG typically was 1–3 mm in thicknesses, which will normally result in a large percentage of consumption of the mechanical energy inputed. Therefore, this PENG was not conducive for small‐scale mechanical energy conversion when applied in biomedical systems. In consideration of this drawback, Lee et al. developed a super‐flexible PENG with same principle by using an Al foil (18 µm) as both the substrate and the electrode (Figure 5D). The device was responsive to tiny motions on human face.^35^ In a typical demonstration, the device was driven by blinking motion, generating an output voltage and current of 200 mV and 2 nA, respectively. Thus, it had great potential to be as active biomechanical sensor. Furthermore, a self‐powered device of multilayered structures without number and size restrictions can be easily integrated based on this kind of PENG. The integration will significantly enhance the output performance of the PENG and efficiency of converting the biomechanical energy. For example, when used in the condition of human walking, a 3.2 V and a 0.195 µA of output voltage and current can be reached, respectively.

Many research works have shown us that the vertical ZnO nanowires can be fabricated on various substrates, such as polymers fibers and metals. In 2008, a fiber like PENG for harvesting low frequency vibration/friction energy was fabricated by using vertical ZnO nanowireson fibers. By entangling two fibers and brushing the nanowires rooted on them with respect to each other, mechanical energy was converted into electricity becaus of coupling the piezoelectric–semiconductor process by brushing the nanowires on two entangled fibers (Figure 5A).^36^ This work established a methodology to scavenge body‐movement energy using fabrics, showing the distinct advantages of designing wearable, adaptable and flexible power source for smart applications in personal electronic. Lee et al. also demonstrated a fiber PENG to convert low frequency mechanical energy into electricity, based on ZnO nanowires and a conducting fiber coated by PVDF. The folding‐releasing motion of human elbow can drive this device to produce an output voltage of 0.1V and power density of 16 µW cm^–3^. Similarly, Lee et al. and Li et al. also fabricated a flexible, fiber nanogenerator based on ZnO.^37^ This device demonstrated an output voltage and average current density of 3.2 V and 0.15 µA cm^–2^, respectively. A ultrasensitive sensors for human heart can be composed by this PENG.

### 3.2 Thin Film Devices

#### 3.2.1 PZT Based PENGs

PZT is a traditional piezoelectric material that is preferred by researchers for its higher electromechanical coupling coefficients than many other conventional piezoelectric materials such as BaTiO_3_.^14^, ^31^, ^38^ For a long time, due to its fragile nature, PZT thin film was considered inflexible and not stretchable. A structural failure will be caused by a stretch in slight scale and 0.2% is the maximum safe strain range of PZT. Researchers have proposed various innovative designs of PENGs based on the different morphology and characteristics of PZT to overcome this undesirable property. A transfer printing method was used by Qi et al. to transferred PZT nanoribbons onto flexible rubber or plastic substrates in large scale and high production.^39^ An 1 cm^2^ PENG can generated voltage and current output up to 0.25 V and 40 nA, respectively, which was prepared by printed nanoribbons of PZT. The same group further developed a stretchable PENG by integrated the wavy/buckled PZT ribbons with PDMS thin film to increase ability of strain, which can be stretched up to 8% strain and can facilitate its integration with flexible structure. A PENG consisting of 10 PZT ribbons and a current of 60 pA can be ganerated by a device with ten PZT ribbons.

**Figure 6:**
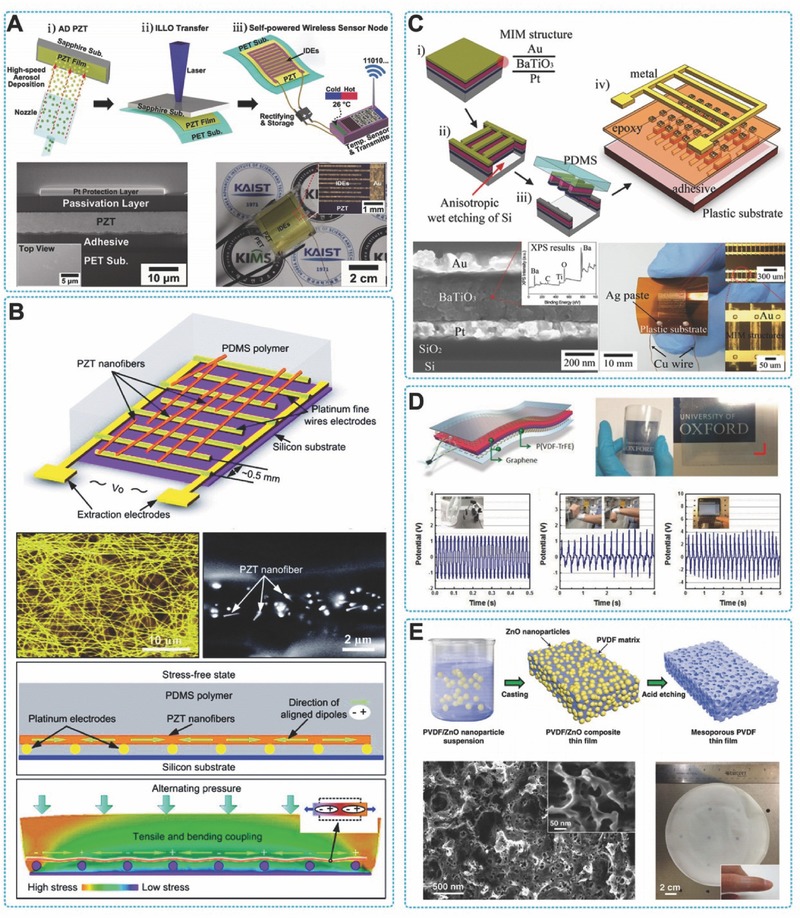
PENGs with thin film structure. A) A schematic illustration of the fabrication process of the flexible and large‐area PZT thin‐film PENG using the ILLO process. Reproduced with permission.^14^ B) Thin‐film PENG based on laterally‐aligned PZT nanofibers. Reproduced with permission.^41^ Copyright 2010, American Chemical Society. C) Flexible BaTiO_3_ thin film PENG. Reproduced with permission.[[qv: 44b]] Copyright 2010, American Chemical Society. D) Thin film (P(VDF‐TrFE)) based PENG on flexible substrates. Reproduced with permission.^51^ Copyright 2011, Elsevier. E) Porous PENG based on PVDF film. Reproduced with permission.[[qv: 49b]]

Park et al. reported a large‐area and highly efficient, PENG with PZT thin‐film on flexible substrates. An inorganic‐based laser lift‐off (ILLO) process was used in this method.^40^ The size limitation of PZT thin film was eliminated by the ILLO process, resulting in an output increase of the flexible PENG (**Figure** **6**A). Additionally, the excimer laser and the dry‐type transfer technique advanced the possibility of commercial fabrication of PENG, comparing with other etching method. The large area roll‐to‐roll process and laser irradiation were applied in display industry such as of low‐temperature poly‐silicon (LTPS). During a periodical process of bending and releasing, this device can reach up to ≈200 V in voltage and 150 µA cm^−2^ in currant, respectively, representing an excellent improvement comparing with other flexible PENGs. A 3.5 cm × 3.5 cm large‐area PENG was reported to generate a 250 V of voltage and an 8.7 µA of current, just being bended by a tiny motion of fingers.^14^ This PENG was fabricated by the ILLO process and could lighten 105 LEDs. By applying graphene as the electrodes, a flexible and semi‐transparent PENGs was demonstrated by Kwon et al. The output performance of voltage, current density and power density were ≈2 V, ≈2.2 mA cm^−2^ and ≈88 mW cm^−3^, respectively.

PZT nanofibers are applied to develop high output PENGs as well. A high‐performance PENG was demonstrated by Cheng et al. fabricated by PZT nanofibers of laterally‐aligned structure.^41^ The PZT electrospining nanofibers was ≈60 nm in diameter and ≈500 µm in length, which can generate a 1.63 V and 0.03 µW of output voltage and power, respectively (Figure 6B). Qin et al. prepared a high output PENG fabricated by electrospun ultra‐long PZT nanofibers.^42^ They demonstrated the capability of this integrated PENG by bending, stretching, or twisting into a large degree and there was not damage to the structure of PENG. The output of voltage and current produced by this PENG were 209 V and 53 µA, respectively. The reported PENGs have presented a particularly efficient for electrical‐to‐mechanical energy conversion that demonstrate a promising improvement toward the realization of self‐powered systems for wearable and implantable electronics.[[qv: 42b]]

The PZT based implantable energy harvesting devices can also be powered by ultrasonic, which is an effective way of converting acoustic energy into electricity.^44^ Shi et al. reported a piezoelectric ultrasonic energy harvester (PUEH) based on microfabricated PZT diaphragm array with a size of 5 mm × 5 mm, which has advantages of extra wide operation bandwidth and controlled outputs.[[qv: 43c]] The output performances can be maintained under an appropriate level by adjusting new ultrasound frequency. For instance, when changing the frequency of an ultrasound with 1 mW cm^–2^ intensity from 250 kHz to 240 kHz, the output power density of PUEH can be increased from 0.59 µW cm^–2^ to 3.75 µW cm^–2^ at 1 cm distance away from the ultrasound source. This type of device has shown a great potential as power source for battery‐less implantable medical devices and systems.

#### 3.2.2 BaTiO_3_ Based PENG

BaTiO_3_ has earned attention for its outstanding ferroelectric properties and biocompatible characteristics which are crucial for biomedical applications.^45^ A flexible perovskite BaTiO_3_ thin film PENG was reported by Park et al. Piezoelectric BaTiO_3_ thin film was deposited on a Pt/Ti/SiO_2_/Si substrate by radio frequency magnetron sputtering and polarized between a high electric field (100 kV cm^–1^) (Figure 6C). This BaTiO_3_ ribbons based flexible PENG can produce a 1 V of output voltage and 7 mW cm^–3^ of power density, when bended periodically. To achieve higher flexibility and large‐area fabrication, BaTiO_3_ nanoparticles were utilized to fabricate PENGs. Park et al. mixed the BaTiO_3_ nanoparticles and carbon nanomaterials in PDMS to gain a piezoelectric nanocomposite (p‐NC), which was spin‐coated onto substrates of flexible plastic and cured under a selected temperature.^13^ After electrods deposition and high‐voltage polarization, electric signals were generated from the device by mechianical bending or human body movements. Kim and his co‐workers developed a “layer‐by‐layer” method (LBL) to synthesize BaTiO_3_ nanoparticles (BTONP) based piezoelectric thin film. A multilayer nanocomposite was prepared by oleic acid (OA) ligands stabilized BaTiO_3_ nanoparticles (>20 nm in diameter) and polymers which was functionalized by carboxylic acid (COOH), such as poly(acrylic acid) (PAA). The high affinity between the BTONPs and the ‐COOH groups helped the organizztion of the OA‐BTONP/PAA nanocomposite. By altering the bilayer number, OA‐BTONP size or inserted polymer type, the performance of this piezoelectric and ferroelectric thin films can be precisely controlled. Assembled by LBL assembling method in nonpolar solvent media, the quantity of adsorbed OA‐BTONP could be effectively increased and significantly increased the output power of PENG.

Moreover, a bio‐inspired flexible PENG based on BaTiO_3_ was presented in 2013.^46^ In this work, anisotropic BaTiO_3_ nanocrystals were deposited on a viral template via the metal ion precursors self‐assembling. The filamentous virus provided template for the deposition of an anisotropic, entangled, highly crystalline BaTiO_3_ nanostructures. This flexible nanogenerator based on virus‐enabled piezoelectric structure can generate electricity of ≈6 V and ≈300 nA without additional structural stabilizers, indicating the high output related to the importance structure in nanoscale. This method of using bio‐template is facile and with particularly enlightening meanings for flexible PENG designing and applications.

#### 3.2.3 PVDF‐Based PENGs

PVDF and its copolymer P(VDF‐TrFE) are polymeric piezoelectric material, with high piezoelectric coefficient (d33 = 32.5 pC/N). Its flexibility, transparency, adequate mechanical strength, and high chemical resistance present many advantages in piezoelectric relative applications. Chemical stability and biocompatibility of polymers is beneficial to the biomedical application for in vivo biological sensors and energy harvester. However, a disadvantage should be noticed that to achieve a good performance the aligning of mechanical stretching with the dipoles of β‐phase PVDF is required. There have been proposed various strategies to fabricate flexible PENGs based on PVDF and its co‐polymers. Chang et al. place piezoelectric PVDF nanofibers on easily acquired substrates to direct‐write thin film device by utilizing a near‐field electrospinning (NFES) and then processed with electrical poling.^47^ Under mechanical stretching, the PENG has shown consistent and repeatable electrical outputs voltage of 30 mV and current of 3 nA, respectively. The as synthesized nanofibers were arranged in either serial or parallel connections to form thin film like structure in order to augment the total output. In another case, Hansen et al. used electrospinning technique to synthesize and pattern an aligned nanofibers arrays with two electrodes, following with an in‐plane poling process.^48^ The PVDF nanofibers were oriented by packaged in PDMS to form a flexible thin film device. As the deforming of the device under alternating compressive or tensile force, it can produce output up to 20 mV and 0.3 nA respectively. Sun et al. reported a method to convert the energy from low‐speed airflow to electricity by resonant oscillation of piezoelectric PVDF microbelts.^49^ The electrical energy generated by this PVDF based PENG from low speed airflow was sufficient to operation low power consumption electronic devices, demonstrating its capabilities for the energy harvesting between inhalation and exhalation process.

In order to enhance the output performance, PVDF films with well controlled nanostructures were desired. Nanoporous PVDF based PENG was developed to address this issue.^50^ Cha et al. presented a porous PENG based on PVDF which was fabricated by a ZnO nanoparticle‐assisted preparation method. This nanogenerator with porous PVDF thin film produced an enhanced piezoelectric energy with power density of 0.17 mW cm^–3^, which was 5.2 times and 6 times increase of output voltage and current than the PVDF nanogenerators of bulk materials. In 2014, Mao et al. reported a large‐area sponge like PENG based on the porous PVDF thin‐film.[[qv: 49b]] This wafer‐scale porous PVDF thin films were fabricated by a simple casting‐etching process (Figure 6E) and can be directly integrated into an electronic device, such as a cell phone, to convert environmental energy to electricity. Thier method provide a promising technique for integrating self‐powered electronics. Recently, Cho et al. reported a high performance P(VDF‐TrFE) based PENG through a surface morphology engineering using solvent annealing method for simple and cost‐effective fabrication at room temperature (Figure 6D). This surface morphology engineered PENG presented 8 times enhanced output voltage and current because of well‐aligned electrical dipoles.^51^

In addition to these aforementioned materials, many piezoelectric materials, such as GaN,^52^ PMT‐NT,^53^ 2D‐MoS_2_ 54 and various of their composites,^55^ are possessing special properties to be applied in various flexible devices. This technique of PENG is scalable and integratable to fabricate practicable and advanced biomedical electronics for healthcare.

### 3.3 Triboelectric Devices

#### 3.3.1 Materials

**Figure 7:**
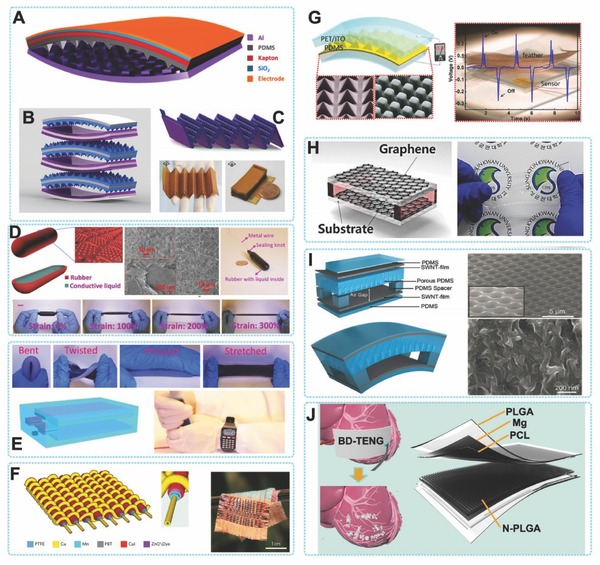
Device structure design and material selection of flexible TENGs. A) The typical arch shaped TENG. Reproduced with permission.[[qv: 22b]] Copyright 2012, American Chemical Society. B) Stacked arch‐shaped TENGs. Reproduced with permission.^67^ Copyright 2013, Elsevier. C) Zigzag TENGs. Reproduced with permission.^68^ Copyright 2013, American Chemical Society. D, E) Stretchable TENGs. Reproduced with permissions.^72^, ^73^ Copyright 2016, the American Association for the Advancement of Science and the American Chemical Society. F) Fiber shaped TENG. Reproduced with permission.[[qv: 73b]] Copyright 2016, The Nature Publishing Group. G) Transparent polymer based TENG. Reproduced with permission.^63^ Copyright 2012, the American Chemical Society. H) Graphene based TENG. Reproduced with permission.[[qv: 56a]] I) CNT based TENG. Reproduced with permission.^58^ J) Bio‐degradable TENG.

The material properties including friction, work function, electron affinity and so on, play important roles in TENGs' output performance. Basically, nearly all materials can exhibit triboelectricity. So, pairing the right materials can achieve output in maximum. The triboelectric series is a guidance,^17^ in which the capability of a material to gain or lose an electrons is shown as a qualitative indication. As we know, almost all kinds of materials, such as metal, polymer, silk and wood, have triboelectrification effect. Thus, they are all potential slections for producing TENGs. In consideration of some other aspects, such as mechanical properties, stability and biocompatibility, a variety of commercially purchased organic materials (e.g. PTFE, PET, PI, PDMS, PMMA) and inorganic materials (e.g. ITO, Al, Cu, Au, Ti, TiO_2_, Si) have been utilized for fabricating TENG and showed outstanding performance.^16^, ^19^, ^56^ Recently, various of advanced materials also have been studied, including graphene,^57^ carbon nanotube,^58^ paper,^59^ nano‐Ag ink,^60^ degradable polymers etc^61^ (**Figure** **7**).

TENG based on carbon‐based materials have been reported recently (graphene, carbon nanotube, etc.). The conductivity, stretchability, triboelectric properties, friction and their roughness in nano‐scale might facilitate the application in TENG.^62^ The graphene‐based TENG (G‐TENG) was reported by Kim et al.[[qv: 56a]] By transfered layer by layer, different number of layers of graphene (from 1–4 layers) were stacked for fabricating the TENG (Figure 7H). It was revealed that the output voltage and current was decreasing with the increasing of graphene layers. For example, the current density were 500, 250, 160, and 100 nA cm^−2^ corresponding to the layers of graphene from 1, 2, 3, and 4 in the G‐TENGs. The different electronic interactions between stacked graphene layers were interpreted as the main reason. A roll‐to‐roll method was applied to produce the G‐TENG by Liu et al. to improve the fabrication proces.[[qv: 56b]] A CNT‐based TENG was developed by Bao et al. (Figure 7I), which could generate tens of volts and tenths to several µA cm^−2^ of output voltage and current density.^58^

As for some environment and human friendly considerations, biodegradable materials were also employed for TENG construction. Kim et al. applied silk fibroin film to build a biocompatibility and eco‐friendly bio‐TENG. The output performance of bio‐TENG was 16 V for voltage and 2.5 µA for current.[[qv: 60b]] Zheng et al. tested the triboelectrical properties of various degradable polymers and based on this result they fabricated fully biodegradable TENG (BD‐TENG) (Figure 7J). The BD‐TENG was implanted in sub dermal region for biomechanical energy harvesting. After a period of implantation, the BD‐TENG can be dissolved in body leaving without any residue. Paper was also involved in the fabrication of TENG for its popular, cheap, lightweight, disposable, and environmentally friendly.[[qv: 60a]]

Structure change will lead to an improvement of the output. Fan et al. studied the influence of varies micro‐fabricated surface structures of PDMS on TENG's output, and found that the TENG with pyramid shaped surface structure can produced highest output compared with that with cubic or liner shaped surface structure.^63^ Recently, Zhao et al. developed a very simple and rapid material surface processing method which can efficiently create liner structure on many friction films and result in an increased electrical output.^64^ Besides, surface charging and chemical modification for the friction material have also been proposed by researchers to not only improve the output performance of TENGs, but also increase their stability in various environmental conditions.^65^

#### 3.3.2 Device Structure Design

Biomechanical energy is small scale, randomness, and multi‐directional. Therefore, TENGs used for biomechanical energy harvesting usually need to be flexible for shape‐adaptive energy harvesting. In 2012, an all‐polymer based flexible TENG with characteristics of simple, cost‐effective for mechanical energy harvesting was firstly demonstrated, through a coupling of triboelectrification and electrostatic induction.^63^ The TENG consisted of two different polymer films with metal electrodes deposited on their back sides. An output voltage and a power density of 3.3 V and 10.4 µW cm^−3^, respectively, was generated by this typical TENG. This most basic working mode of TENG was later defined as vertical contact‐separation mode (Figure 3A).

Since the first demonstration of TENG, the arch shaped structure has become one of the most popular structure due to its simple fabrication, high performance, and universal feasibility.[[qv: 22b,65]] In this structure, TENG with an arch‐shaped gap between a polymer film and a metal foil was fabricated (Figure 7A). In a typical demonstration, the TENG with arch‐shape structure had the *V*_oc_ and *I*_sc_ about 230 V and 94 µA, respectively. The energy conversion efficiency reached up to 39%. Inspired by this structure and its working mechanism, various derived structures are proposed. For example, arch‐shaped and anti‐arch‐shaped TENGs were consisted to alternatively‐stacked structure and electrically connected in a parallel configuration, which has been proved that can significantly enhance the output performance (Figure 7B).^67^ The zigzag structure was another variation of the stacked arch‐shaped structure (Figure 7C).^68^ A device with a 6‐layered construction can generate a current of 656 µA. TENGs with spacer layer is another deformation structure of arch shape. Instead of utilizing the bending of the material itself to form an arch shape gap, a spacer was introduced between two friction layers, which brought a stable gap to TENG. The gap width was determined by the thickness of spacer layer, so its controllability and durability were improved a lot. The existence of spacer facilitated the versatile design of TENG based systems, because shape and the thickness of TENG can be easily controlled.[[qv: 22a,68]] The lateral sliding mode in plane was designed as another mode of the flexible TENGs (Figure 3B). Two films with complementary micro‐sized linear grating arrays were fabricated to a thin‐film‐based micrograting triboelectric nanogenerator (MG‐TENG), which can convert the mechanical energy into electricity from the motion of relative sliding between two micrograting films.^70^ The output current and the output power of the device were up to 10 mA and 3 W, respectively, with the conversion efficiency of ≈50%. In order to harvest mechanical energy in differernt directions, a TENG with a sandwiched structure and checker‐like electrodes was designed by Xi et al.^71^ This TENG generated a voltage of 210 V and showed great performance in either sliding directions. Furthermore, they also demonstrated to light up LEDs by the mouse operation energy when integrated the sliding mode TENG into a mouse pad or sliding panel.

Considering the rapid development of deformable and stretchable electronics, a power source structural and functional suitable for this class of electronics was needed. Yi et al. reported a method for TENGs and highly deformable and stretchable self‐powered sensors.^72^ A shape‐adaptive triboelectric nanogenerator (saTENG) unit with conductive liquid as electrodes can be used to harvest various mechanical energy effectively in different motions (Figure 7D). The saTENG can sustain a 300% strain in maximum and be adapted to almost all of the curvilinear and three‐dimensional surface, beacause of its admirable flexibility. It increased the possibility of employing the saTENG to be wearable energy sources or self‐powered device for sensoring human body motion. A stretchable supercapacitors was combined with a stretchable TENG to develop a stretchable, soft and fully packaged self‐charging power system by the same group.^73^ Because of the fully soft structure, this system can subject to large‐degree deformation and convert energy from various motion, including human body movement (Figure 7E). This self‐charging power system shows wide applications to harvest all kinds of biomechanical energy and fabricate personal wearable self‐powered electronics.

Fiber‐based triboelectric generator (FB‐TENG) have the advantage of harvesting the biomechanical energy from human motion in three‐dimensional.^74^ A low cost and facile FB‐TENG was introduced by Zhou et al.[[qv: 73a]] This wearable TENG is capable of scavenging energy from human motions and body vibration to electric power by using carbon nanotubes, polytetrafluoroethylene (PTFE) aqueous suspension and cotton threads. The power density can reach up to ≈0.1 µW cm^−2^. Recently, Chen et al. have reported a micro‐cable power textile for simultaneously harvesting energy from ambient sunshine and mechanical movement (Figure 7F).[[qv: 73b]] Solar cells fabricated from lightweight polymer fibres into micro cables were then woven via a shuttle‐flying process with fibre‐based TENG to create a smart fabric. A single layer of such fabric was 320 µm thick and can be integrated into various cloths, curtains, tents and so on. This hybrid power textile, fabricated with a size of 4 cm by 5 cm, was demonstrated to charge a 2 mF commercial capacitor up to 2 V in 1 min under ambient sunlight in the presence of mechanical excitation, such as human motion and wind blowing. The textile could continuously power an electronic watch, directly charge a cell phone and drive water splitting reactions. Wang et al. demonstrated a Fiber based TENG for continuously power wearable electronics only by human motion, realized through with optimized materials and structural design. Fabricated by elastomeric materials and a helix inner electrode sticking on a tube with the dielectric layer and outer electrode, the TENG has desirable features including flexibility, stretchability, isotropy, weavability, water‐resistance and a high surface charge density of 250 mC m^–2^. With only the energy extracted from walking or jogging by the TENG that is built in outsoles, wearable electronics such as an electronic watch and fitness tracker can be immediately and continuously powered.[[qv: 73c]]

## 4 Recent Progress in Biomedical Applications

### 4.1 Power Source for Biomedical System

**Figure 8:**
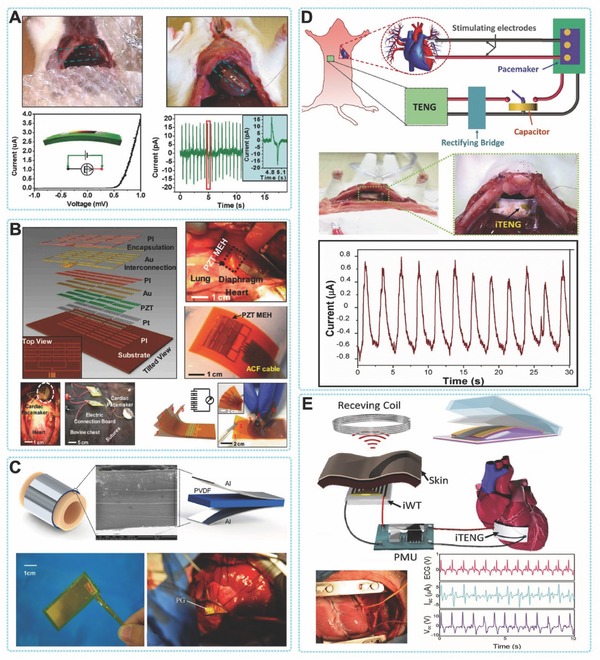
In vivo energy harvesting by PENGs and TENGs. A*)* The first demonstration of in vivo biomechanical‐energy harvesting using a single nanowire based PENG. Reproduced with permission.^76^ B) Conformal energy harvesting from heart/lung by PZT based PENG. Reproduced with permission.^31^ Copyright 2014, National Academy of Sciences. C) Flexible PVDF based PENG for harvesting energy from ascending aorta. Reproduced with permission.^78^ Copyright 2015, Elsevier. D) The first demonstration of implantable TENG for harvesting biomechanical energy.[[qv: 68b]] E) Wireless cardiac monitoring system powered by iTENG.^82^

The recent development of biomedical systems, especially the microelectronic devices for healthcare and other medical applications illustrates the potential of such a futuristic concept.^75^ Some possible applications are exciting and extensive, but they still need reliable and safe energy sources to operate. Considering the application in biology and medicine, batteries can be dangerous, bulky, and difficult to change. For example, replacing a pacemaker battery is alway problematic for causing additional pain and cost. However, great progress has been made in energy harvesters over the past decade. In human body, where there is little light and there are only small thermal gradients, mechanical energy harvesting is an ideal method to provide power for implantable medical devices. Biomedical systems are consuming less and less power, and new harvesting technologies like PENGs and TENGs have the potential to supply the power needed for the safe operation of medical devices (**Figure** **8**).

Harvesting mechanical energy using PENG has been demonstrated some time ago. The continuous optimization in flexibility and output performance of the piezo‐devices made them possible for biomedical applications. In 2009, Yang et al. converted biomechanical energy from muscle movement into electricity by a ZnO based PENG.[[qv: 27b]] This device was a single nanowire generator that consisted of a lateral ZnO nanowire with two ends affixed on a flexible substrate. Muscular movement drove the back and forth deformation of the whole device. The electrons flowing in the external circuit was launched by the piezoelectric potential generated generated inside the ZnO nanowires. By integrating multiple nanowires, the output *V*_oc_ can be increased and four ZnO nanowires in a series connection can produced an output voltage of 0.15 V. Scavenging low‐frequency energy from a running hamster or a tapping finger was successfully demonstrated revealing the potential application for harvesting regular and irregular biomotion by nanogenerators. Based on the same mechanism, Li et al. developed an implantable PENG based on single ZnO nanowire to harvest energy in breath and heartbeat in a living rat.^76^ This is the first demonstration of harvesting in vivo biomechanical‐energy by an PENG (Figure 8A). Typically, the average magnitude of the in vivo voltage and current signals were about 1 mV and 1 pA, respectively. Besides energy harvesting, some crucial results for in vivo application were also addressed in this work, such as the biocompatibility, toxicity, encapsulation, anchoring and the potential for active sensing of in vivo biological motions. This study not only shows the potential of scavenging in vivo mechanical energy by PENG, but also starts a new trend for pursuing sustainable power source of implantable medical devices. Although such a model highlights the potential for self‐powered biomedical devices, there is still an important practical challenge in the efficient output power. Dagdeviren et al. utilized a thin‐film PZT nano‐ribbon based PENG for conformal energy harvesting and storage from motions of the diaphragm, lung, and heart. Figure 8B presents schematic diagrams of the flexible PZT thin‐film PENG.^31^ A bridge rectifier and a micro‐battery can be integrated with the flexible PZT mechanical harvester on a flexible substrate. The device was further packaged by biocompatible encapsulation layer to harvest energy directly from the movement of bovine heart and lung in vivo. During the cardiac contraction and relaxation, the conformal contact of the whole device with the heart without any destruction was significant importance for in vivo applications of energy harvester. The output *V*_oc_ was up to 5 V and greater by three orders of magnitude than previous in vivo results, when the in vivo PENG was attached on the right ventricle (RV) of a bovine heart. In this work, simultaneous energy harvesting and storage were achieved by fully integration of PENG, rectifiers and micro‐batteries.

Thier research indicates strong feasibility of achieving self‐powered implantable biomedical devices. This work also provides an interesting demonstration of five independent PZT harvesters connected serially in a multilayer‐stacked flexible PENG. This multilayered flexible PENG could generate an 8.1 V of voltage and 1.2 µW cm^−2^ of power density, which were much higher than the output of the single‐layer device. In 2015, a similar work was presented by Lu et al.^77^ They developed an ultra‐flexible PZT based energy harvester, which had the similar structure of above‐mentioned device. It can harvest the mechanical energy derived from cardiac motions by integrated with the heart. For simulating the scenario of applying mechanical energy harvester in body, in vivo tests were performed in differernt settings of the experimental animals, for example opened/close chest, awake and under anesthesia. When the PENG was fixed from left ventricular apex to right ventricle, this ultra‐flexible piezoelectric device could generate a 3V voltage, which demonstrated the potential applications of harvesting the in vivo mechanical energy from cardiovascular system and body motion for powering the implantable medical devices and sensors sustainably. Apart from the biomechanical energy that can be easily through of, such as human motion, heartbeat and breath, some special tissues or organs also contains a wealth of mechanical energy. However, harvesting this kind of mechanical energy needs more sophisticated design of devices. Zhang et al. reported a PVDF based flexible and implantable PENG, which wrapped around the ascending aorta and scavenged the energy from the pulsation.^78^ This research reveals the in vitro and in vivo studies of harvesting the energy in artery pressure with a flexible and implantable PENG (Figure 8C). In the in vitro study, the max output power (P_max_), voltage (V_max_) and current (I_max_) and of the PENG were 681 nW, 10.3 V and 400 nA respectively. The quantity of electric charging by one pulse was about 7–9 nC. When implanted the PENG in vivo by wrapping around the ascending aorta of a porcine, the V_max_ and V_max_ of the implanted PENG were 1.5 V and 300 nA under the heart rate of 120 bpm and the blood pressure of 160/105 mm Hg. The instantaneous output power could reach up to 30 nW with a long‐lasting duration of 700 ms and 77.8% duty ratio. The implanted PENG could charge for a 1 µF capacitor to 1.0 V within 40 s.

Currently, in order to improve the efficiency of energy conversion, TENGs were introduced for biomechanical energy harvesting. Since 2012, a series of work reported that energy from human motion has been successfully collect under in vitro conditions by TENG, such as walking, running, finger tapping or elbow bending, which demonstrated its potential for biomedical applications.^79^ In 2014, Zheng et al. first demonstrated the in vivo application of TENG.[[qv: 68b]] In this work, an implantable triboelectric nanogenerator (iTENG) was implanted in a living rat to harvest energy from its periodic breathing (Figure 8D). The iTENG was fully encapsulated to protect its inner structure from physiological environment. The implantation site was near the chest, which allowed the iTENG to convert the mechanical energy from the normal inhalation and exhalation of rats into electricity and used to power a prototype pacemaker. Through theoretically calculation, the energy harvested during 5 breaths can generate a single pulse to stimulate the rat's heart. This was a significant progress of charging an implanted medical devices by a TENG. Shi et al. reported a packaged self‐powered unit based on piezoelectric and triboelectric hybridized nanogenerators (PTNG).^80^ The PTNG was fabricated with a BaTiO_3_@PDMS film as a contact layer based onvertical contact‐separation working mode. The polarized BaTiO_3_@PDMS film was bended to generate a piezoelectric potential and contacted with aluminum foil to generate a triboelectric effect simultaneously. Both of the piezoelectric potential and triboelectric effect was coupled and increased the output performance of the PTNG. The power density of the PTNG was increased by 80% and reach up to 97.41 mW m^–2^, compared with the TENG in control. The PTNG was further integrated with several power management components to form a packaged self‐powered system. The self‐powered system was utilized to harvest and store the biomechanical energy, and then to successfully power a temperature senser that was implanted in vivo. It has to be noted that, the entire packaged self‐powered system was a viable “Plug and Play” mobile energy source by designing the universal connections of plug and socketfor the system.

Tang et al. demonstrated that flexible TENGs attached to human articulations could generate enough electricity power a medical laser.[[qv: 78c]] The aim was to use this laser to accelerate bone remodeling treatments. Such a flexible TENG does not have the same ability to power a device over time compared to a battery, because the specifications and behaviors of TENGs are different: batteries deliver continuous power until they are exhausted, while flexible TENGs deliver a pulse of power every time they are activated. Producing a continuous light emission during 60s when powered by a battery was equal to a series of 100 light pulses when powered by a flexible TENG. Tang et al. showed that even if the TENG powered laser was a little less efficient than the battery‐powered laser, both treatments can successfully accelerate bone remodeling. This finding is important because it proves that TENGs with a small energy buffer such as a capacitance can be sufficient to power some small biomedical devices.

Recently, Zheng et al. developed a reliable encapsulation method of TENG, which provide the TENG outstanding stability in harsh environment.^81^ Base on this technology, the same group designed a novel iTENG with significantly improved in vivo output and reliability.^82^ A ‘*Keel structure*’ was introduced to facilitate the contact and separation process of TENG in complicated environment of living body (Figure 8E). Driven by the heartbeat of an adult Yorkshire porcine, the *V*_oc_ and *I*_sc_ can reach up to 14 V and 5 µA, which were improved by factors of 3.5 and 25, respectively, compared with the reported in vivo output performance of PENG and TENG. The in vivo performance of the iTENG was evaluated for over 72 h of implantation, revealing the outstanding stability of iTENG for generating electricity continuously in a living animal. The iTENG was then applied to power a wireless transmission system (SWTS) and the in vivo related signals of heartbeat was successfully transmitted, showing the feasibility of iTENG for powering the electric medical sensors in mobile and real‐time wireless monitoring.

### 4.2 Active Pressure/Strain Sensor

The human‐body activity induced pressure is distributed in a large range from a low‐pressure regime to a high‐pressure regime (10–100 kPa).^83^ Physiological activity in different positions of human body will generate differeent pressure signals. For instance, intraocular pressure and intracranial pressure can belong to the low pressure regime. The medium‐pressure regime is mainly generated by the respiration motion, heart beating, blood pressure wave, jugular venous pulse, radial artery waves and phonation vibration. The body weight brings the high pressure regime under the feet. The monitoring of these pressures by suitable pressure sensors is important for the application in diagnostics of heart failure, cardiovascular disease, respiratory disorders and damaged vocal cords. It was also crucial for monitoring the sleep apneahyponea syndrome, sports injuries in athletes and high‐risk diabetic foot ulceration. For developing high sensitive, durable, stretchable and flexible pressure sensors, piezoelectric and triboelectric materials and devices have been paid much attention to, recently. According to the structures and materials of the active sensors, each of them has its own advantages in sensing mechanisms.

#### 4.2.1 Active Pressure Sensors

**Figure 9:**
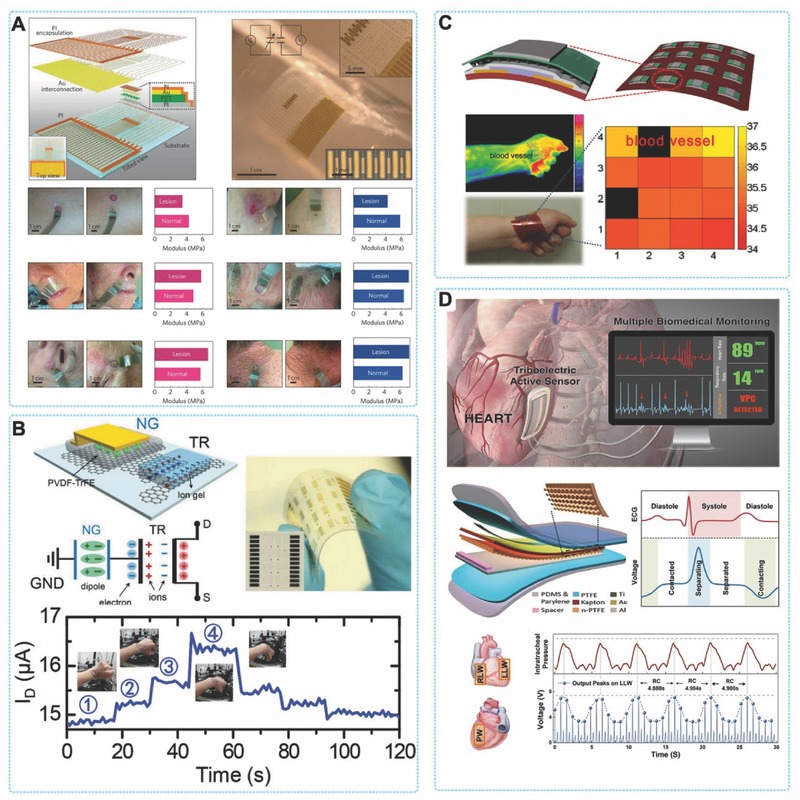
Active pressure/strain sensor based on PENG and TENG. A) Conformal modulus sensor based on PZT. Reproduced with permission.^90^ Copyright 2015, Nature Publishing Group. B) Active‐matrix strain sensor based on a PENG‐powered graphene transistor. Reproduced with permission.^96^ C) Structure of an OFET composed of microstructured P(VDF‐TrFE). Reproduced with permission.[[qv: 83a]] D) Implantable active sensor based on TENG. Reproduced with permission.^92^ Copyright 2016, American Chemistry Society.

PENG based pressure sensors is fabricated and the generated electric charge is proportional to the applied pressure when a piezoelectric material is stressed. For the development of a flexible piezoelectric pressure sensor, PVDF and its co‐polymer P(VDF‐TrFE) are ideal candidate materials for their flexibility and the ease of the fabrication.^84^ Although inorganic materials have inferior flexibility comparing with organic materials, various inorganic materials also exhibit distinct mechanic flexibility when processed into ultrathin films or nanowires. Therefore, ultrathin films or nanowires based on piezoelectric inorganic materials have been attracted much attention and researched comprehensively for flexible and stretchable pressure sensers. A PENG based pressure sensor was reported by Zirkl et al. and Graz et al., which was integrated with an amplifying element of transistor for reading the signal from the sensor.[[qv: 83b,c]] The sensing element was a PENG based on P(VDF‐TrFE) or P(PVDF‐TrFE)/PbTiO_3_ nanocomposite. When applied a pressure on the PENG integrated sensor, it responsed a voltage signal. This voltage signal from PENG controlled the magnitude of current in the integeated transistor and swiched on/off the gate of the transistor. However, some limitations of this pressure sensors with an amplifying element of transistor have to be noted here. The fabrication process is more complicated and the energy consumption is high, which are caused by the additional inner connections of PENG and transistor element. Therefore, another approach of employing a piezoelectric polymer direct integrated into a transistor as a gate dielectric layer is demonstrated in presure sensors. Many groups, such as Tien and co‐workers,[[qv: 83a,84]] Kim et al.,[[qv: 83d]] and Trung et al.,^86^ directly coupled a P(VDF‐TrFE) and P(VDF‐TrFE)/BaTiO_3_ nanocomposite as gate dielectric layers with an organic field‐effect transistor(OFET) (**Figure** **9**C). The equivalent voltage or remnant polarization inside the piezoelectric polymer was changed by the direct piezoelectric effect under external force. Meanwhile, the applied external force also led to a change of source‐drain read‐out current by modulating the intensity of the charge that accumulated between the semiconductor/dielectric interface.

In addition, Persano et al. introduced a high‐performance pressure sensor, which was fabricated by P(VDF‐TrFE) nanofibers.^87^ By the electrospinning method, a large‐area free‐standing P(VDF‐TrFE) fibers was achieved in a flexible PENG. This as fabricated PENG based sensor showed exceptional piezoelectric characteristics with a high sensitivity to pressure, which could monitor small pressure about 0.1 Pa in minimum. These progress displayed the wide potential application of this active pressure sensor from self‐powered micro‐mechanical elements to sensitive impact bio‐detectors. Cheng et al developed a thin film PENG based on PVDF for implantable and self‐powered monitoring of blood pressure.^10^ An excellent linear relationship was achieved between the output voltage of device and blood pressure, with a high‐sensitivity of 173 mV/mm Hg, which was significantly improved than reported results.^88^ The device also showed excellent stability for over 50,000 operating cycles. An in vivo experiment in adult Yorkshire porcine showed a sensitivity of 14.32 mV mm^–1^ Hg. Based on these characteristics of the device, they established an implantable, self‐powered and visualized blood pressure monitoring system.

A PZT conformal pressure sensor was introduced by Dagdeviren et al.^89^ A sheet structure of PZT array was connected with the gate electrodes of a metal‐oxide‐semiconductor field‐effect Transistor (MOSFET) to form a stretchable, lightweight and ultrathin devices. This device showed a fast response time of less than 0.1 ms, a high detection resolution with detection limit of 0.005 Pa, high stability and high sensitivity. After stretched at 30%, there was no impact to the performance of the sensor. This sensor was also softly laminated on the wrist, neck and throat to monitor human blood pressure. A conformal modulus sensor (CMS) with ultrathin, stretchable networks of sensors and mechanical actuators based on PZT was introduced by the same group as well (Figure 9A).^90^ The sophisticated sensor demonstrated to measure the viscoelasticity of the epidermis in the surface regions. These conformal avctive sensor systems can be compactly attached with soft tissues, organs, and various biological substrats of live creatures, which facilitate their biomedical applications.

Since the first proof‐of‐concept demonstration, the TENGs have been proved to be one of the most convenient way to demonstrate the pressure sensing capabilities. Such self‐powered active sensor can detect gentle external mechanical force like a 20 mg falling feather with 0.4 Pa in contact pressure and a 8 mg water droplet with 3.6 Pa in contact pressure through relative deformation between two sheets of polymer by pressure. For biomedical pressure sensing, the devices should be deformed to adapt the complex shapes of human body and even soft biological tissues and organ systems. Yang et al. assembled serpentine‐patterned electrodes and a wavy‐structured polymer film together to develope a TENG with new stracture.^91^ Owing to the unique design, this flexible TENG (FTENG) could be operated at both stretching and compressive movement, which was ideal for strain monitoring. A high‐output power density of 5 W m^−2^ was delivered under the traditional compressive mode. The FTENG could sustain 22% tensile strain under stretching and generate almost 70 times of power output comparing with the TENG of planar structure. For the superior feature of the reliable output performance on curved surfaces, the FTENG could be employed as pressure sensor to detect tiny motions of muscles, joints and prominentia laryngea with close fitting to human skin. This work presents a progress of self‐powered pressure sensing and orients to the new generation of bio‐integrated systems.

Very recently, Ma et al. proposed a self‐powered and multifunctional implantable triboelectric active sensor (iTEAS) based on implantable TENG, which can provide accurate, continuous, and real time monitoring of multiple physiological and pathological signals.^92^ As demonstrated in large‐scale animals, the device can monitor heart rates, reaching an accuracy of ≈99%. So, cardiac arrhythmias such as atrial fibrillation and ventricular premature contraction were successfully detected in real‐time (Figure 9D). Furthermore, this device also provided a novel method of monitoring respiratory rates and phases by analyzing variations of the its output peaks. Blood pressure can also be independently estimated and the velocity of blood flow calculated with the aid of a separate arterial pressure catheter. The in vivo biocompatibility of the device was examined after 2 weeks of implantation, proving suitability for practical use. As a multifunctional biomedical monitor that is exempt from needing an external power supply, the proposed iTEAS holds great potential in the future of the healthcare industry.

#### 4.2.2 Active Strain Sensors

A strain sensor or strain gauge isused to measur the deformation of objects. Usually, a patterned metal foil on a flexible substrate constitutes a typical strain sensor for monitoring deformation on an object.^93^ Recently, according to the huge requirements of electric skin and variouse motion monitoring for rehabilitation assistance and developing diagnosis, the interest of developing flexible and stretchable strain sensors integrated on cloth or human body was increased.

Body motion monitoring of human is of great importance and can be sorted out to differernt categories: large‐scale and small‐scale motions. The large‐scale motions is the movements of limbs and the small‐scale motions is the subtle movements, such as speaking, swallowing and breathing.^94^ These movements are crucial and indicating the conditions, damages and diseases of human body. For example, applying strain sensors to diagnose respiratory disorders, damaged vocal cords and monitoring Parkinson's disease.

Piezoelectric strain sensors rely on PENGs that made of piezoelectric materials, which can convert mechanical energy into electricity. And they have the advantage of low power consumption, high sensitivity and ultrafast response.^95^ Recently, P(VDF‐TrFE) polymer material and Zinc oxide naowires have been used in developing piezoelectric strain sensors.^95^, ^96^, ^97^ An strain sensor with active‐matrix was reported by Sun et al., which was based on a PENG‐powered coplanar‐gate graphene transistor.^96^ Due to the external strain, the P(VDF‐TrFE) generated a piezopotential, which was coupled to the channels of graphene transistor by dielectric ion gel effectively (Figure 9B). The piezopotential modulated the channel conductance of the graphene transistor. This strain sensors showed ultrasensitivity and high flexibility to detect ≈0.008% of minimum detectable strain, which were promising for human‐activity monitoring. ZnO nanowire is semiconductive and piezoelectric material, which is also used to fabricate strain sensors. Under applied tensile strain, a piezoelectric potential and charges were generated between two ends of the ZnO nanowire. The applied external strain can control the height of the potential barrier and adjust the transport behavior of the charge carriers.^98^ A single ZnO piezoelectric fine wire (PFW) was fabricated to build a flexible strain sensor by Zhou et al.[[qv: 96e]] This piezotronic strain sensor showed great stability, high sensitivity, and very short response time. The flexible ZnO PFW strain sensor is of great potential to be applied in measuring cell mechanical stress and strain. In addition, a single ZnSnO_3_ nanowire was developed to a strain sensor with high‐sensitivity by Wu et al.[[qv: 30b]] The sensitivity of a ZnSnO_3_ NW‐based strain sensor was 19 times highter than Si devices and 3 times higher than those of ZnO and CNT nanowires. However, the piezotronic nanowires of ZnO or ZnSnO_3_ are usually attached on the surface or inset the substrat of strain sensors, which brings some disadvantages of detecting strains at discrete points.

Strain sensors were developed based on a flexible substrate with in situ synthesised ZnO nanowires. Vertically aligned ZnO nanowire arrays were fabricated to a strain sensor reported by Zhang et al., which had higher sensitivity compared with the single ZnO nanowire based strain sensor device.[[qv: 96b]] Moreover, another flexible strain sensor was developed on carbon fiber textile with a ZnO nanowire film grown.^99^ Gullapalli et al. demonstrated a piezoelectric strain sensor based on paper matrix with ZnO nanostructures embedded in, for improving the flexibility of entire device.^95^ This strain sensor presented quite low power consumption and great strain sensitivity.

Generally, piezoelectric strain sensors based on nanowire materials showed high sensitivity, fast response, and low power consumption. However, they still have some limitations, especially in flexibility, stretchability, and detection range. These disadvantages limite the applications of piezoelectric strain sensors in measuring curved objects or the irregular surface on human body for monitoring human‐activity, healthcare devices and wearable electronics. A conformal piezoelectric strain sensor with better flexiblity and stretchability is crucial for biomedical applications.

### 4.3 Direct Stimulation of Living Cell, Tissue and Organs

Some illnesses such as chronic pain, abnormal heart rate, and Parkinson's syndromes can be eased or cured by stimulating the spinal cord, heart, and the brain at an in vivo state using electrical pulse.^100^ Most biomedical stimulation devices consumed their embedded battery power providing such functional electrical stimulations for particular muscles and nerves generally.^101^ To solve the energy limitation of electrical medical devices that the flexible PENGs and TENGs generate electric power and supply a functional electrical puls to stimulate muscles and nerves is of prime importance.[[qv: 42a]]

**Figure 10:**
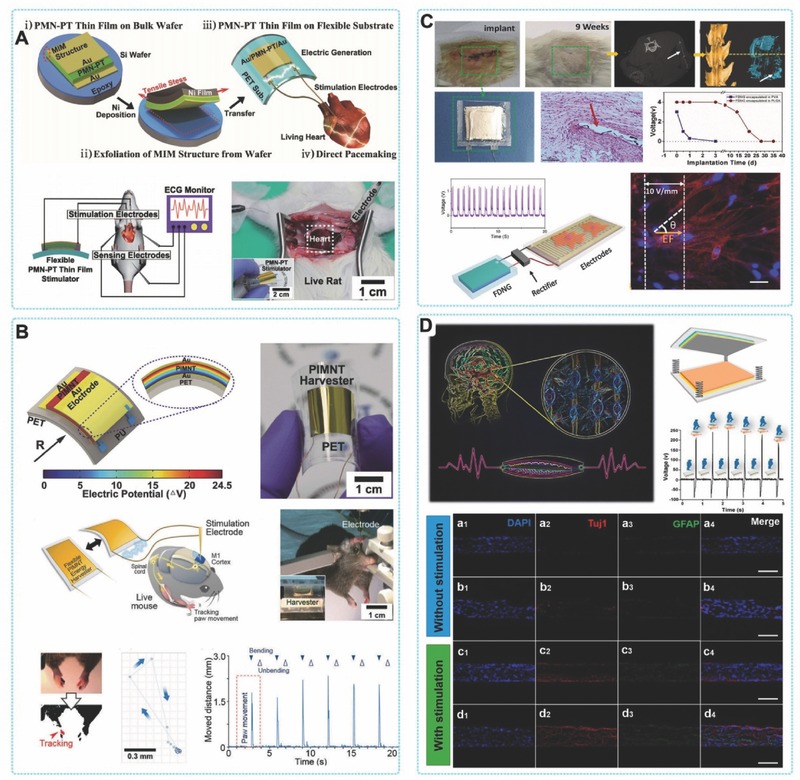
Direct stimulation on cell, tissue and organ by PENG and TENG. A) A schematic of the experimental setup for artificial cardiac pacemaking using the electric output from the flexible PMN‐PT thin‐film PENG. Reproduced with permission.^15^ B) Self‐powered deep brain stimulator using the electric output from thin‐film PENG. Reproduced with permission.^103^ Copyright 2015, Royal Society of Chemistry. C) Electrical stimulation for neuron orientation based on BD‐TENG. D) A self‐powered neural differentiation system with a step‐driven TENG as the electrical simulation power source. Reproduced with permission.^106^ Copyright 2016, American Chemistry Society.

Lately, Hwang et al. proposed a method to use a flexible PMN‐PT thin film nanogenerator to output momentary electric energy and stimulate the heart of a living rat directly (**Figure** **10**A).^15^ The PMN‐PT thin film PENG stimulated the rat heart with electric pulses and the electrocardiogram (ECG) was simultaneously recorded by attached sensing electrods. Figure 10A showed the artificial heartbeat generated with the assistance of PENG in an animal experiment with chest laparotomy. Before stimulating, the the ECG signals showed typical P, T waves, and regular QRS complex with a heart rate of 6 beats per second. A few micro joule of electric power was required for triggering the action potential for artificially stimulating the living heart.^102^ When the flexible PENG was bent and released periodically, corresponding electrical signals were recorded in the ECG with the cardiac bioelectricity of rat. The electric energy generated by the flexible PENG was 2.7 µJ and capable of exciting the artificial cardiac action potential of the rat.

The same group also presented a high‐performance flexible PENG enabled by a single crystalline thin film of Pb(In_1/2_Nb_1/2_)O_3_–Pb(Mg_1/3_Nb_2/3_)O_3_–PbTiO_3_ (PIMNT) on a plastic substrate and used as a self‐powered deep brain stimulator (DBS) in vivo.^103^ A modified Bridgman method with a stress‐controlled nickel exfoliation process was used to grow and transfer thin film of PIMNT onto a flexible substrate, without any mechanical damages. This PENG stimulator can generate a *V*_oc_ and an *I*_sc_ of 11 V and 285 mA, respectively, when bent periodically on a linear stage. These results matched with load impedance for the electrode of practical DBS and were adequate to stimulate nerve nuclei. A maximum output current of 0.57 mA was measured when fixed and bended by a human finger. Meanwhile, the PIMNT thin film PENG could light on 120 green LEDs and easily charge capacitors for the output power was about 0.7 mW. A demonstration of control body motions in a live rat was verified by stimulating the primary motor (M1) cortex. When the PIMNT powered the real‐time DBS to activate M1 cortex with functional electric potential, the muscle contraction and the motion of the forelimb was induced (Figure 10B).

Various electric stimulation technologies were also applied in cell manipulation, which provided an exciting route for tissue engineering and many of them have been proved effective in research and clinical settings.^104^, ^105^ Guo et al. combined a highly electrically conductive rGO−PEDOT hybrid scaffold with a step‐driven TENG as the electrical simulation power source to build a self‐powered neural differentiation system.^106^ The electrical outputs of 250 V and 30 µA were achieved by driving the TENG with human step motion, which was sufficient to stimulate the MSC cells. The rGO−PEDOT hybrid microfiber can not only enhance the proliferation of MSCs but also function as a medium for step‐driven TENG pulse electrical simulation signals, which can induce MSCs to differentiate into neural cells. The study realized an enhancement of MSC neural differentiation on the rGO−PEDOT hybrid microfiber under TENG‐driven electrical pulse simulation. This work shows a significant potential application of a self‐powered TENG electrical stimulation system for the assistance of nerve regeneration (Figure 10C).

Zheng et al. reported a biodegradable triboelectric nanogenerator (BD‐TENG) for short‐term stimulation of neural cells in vivo.[[qv: 60a]] The *V*_oc_ and *I*_sc_ of BD‐TENG were ≈40 V and ≈1 mA, respectively. When two complementary micrograting electrodes were powered by BD‐TENG, an1 Hz and 10 V mm^–1^ DC‐pulsed electric field (EF) was generated. This DC‐pulsed EF oriented the growth direction of nerve cells cultured on the micrograting electrode successfully, which was crucial for neural repair (Figure 10D). Several unique advantages were exhibited by the BD‐TENG here. When it was implanted in vivo, various kinds of biomechanical energy in respiratory motion, heartbeat, and pressure of blood vessels can be converted into electrical power. For example, a BD‐TENG is implanted under the skin of the left thorax of a rat, the inhalation and exhalation of the rat can result in an alternative expansion and contraction of the thorax, which deforme the BD‐TENG and make two fraction layers to contact and separate periodically. During this course, the electric potential caused by the contact electrification and electrostatic induction drives the electrons flowing back and forth in the external circuit along with the respiratory motion. So a continuous AC output is generated with the respiratory movement continues. The satisfactory biocompatibility and light‐weight, cost‐effective, and designable size will further promote the electric potential's in vivo application. The low frequency and the relatively small amplitudes are suitable for in vivo electrical stimulation. Furthermore, fully implantable stimulation or diagnostic devices could be fabricated if integrated with a specially designed electrode or wireless transmission component. The implanted devices can be left behind in the body even when the therapeutic or diagnostic process is completed. The whole device can be degraded and absorbed gradually without any residue. The BD‐TENG revealed many outstanding advantages and tremendous potential as aremarkable power source candidate for transient in vivo medical devices.

## 5 Conclusions and Outlook

Biomedical systems and nanotechnologies are revolutionizing healthcare and medicine; their synergy could be extremely powerful, and they could play key roles in near‐term medical technologies. Taking into account the decreasing power consumption of microchips and the increasing efficiency of nanomaterials‐based mechanical energy harvesters such as PENGs and TENGs, which should be possible to power autonomous biomedical systems. Reaching energetic independence from current bulky batteries is a first important step for their development. However, to reach this purpose, the optimization of the output performance and power management of nanogenerators need to be addressed in order to increase the energy conversion rate and efficiency in use. Furthermore, to realize their full potential in the field of healthcare, nanogenerators need to continue to evolve more high flexibility, sensitivity, elasticity, stretchability, durability and biocompatibility, to be fully operational in the human body. Autonomous biomedical systems with self‐powered and active sensing properties will be the future development direction of the medical field. For easily using ex vivo and in vivo, they need to be wearable and implantable. In order to achieve this, they will need to be fully flexible to fit the shape of organs, including skin, closely. Thus, the devices will be more discrete and comfortable for the patients, and will be better adapted to their targeted tissue/organ, which will increase their sensing ability and the amount of energy harvested.

Flexible electronics are currently used, flexible microelectronics are under development,^107^ and flexible PENGs and TENGs are progressing. Therefore, flexible autonomous biomedical devices are possible, and the challenge will be the integration of all the parts into one small, flexible device. Then the device will need to be embedded into a flexible, biocompatible package to be patched on or implanted into a patient. Improving and facilitating this step is another important goal for biomedical systems.

Fully biodegradable, high‐performance electronics and sensors, defined as transient electronics are another class of novel devices that under fast growing. They are fabricated with biodegradable organic/inorganic materials that are used widely in medical devices. The integration of these devices could expand their functional capabilities in medicine, especially in the cases that a medical device is just need to be temporarily implanted in human body, for example, some bio‐sensors, stimulators and drug delivery systems. Suitable power source that functionally and structurally compatible with transient electronics is of great importance. The newly appearanced BD‐TENG is a potential solution. Future works in this field will focus on the performance improvement, structural integration and optimization and intelligent control of their dynamic properties in vivo, such as the operating lifetimes and the absorption efficiency.
